# Decay and nutrient dynamics of coarse woody debris in the Qinling Mountains, China

**DOI:** 10.1371/journal.pone.0175203

**Published:** 2017-04-06

**Authors:** Jie Yuan, Lin Hou, Xin Wei, Zhengchun Shang, Fei Cheng, Shuoxin Zhang

**Affiliations:** 1 College of Forestry, Northwest A&F University, Yangling, Shaanxi, China; 2 Qinling National Forest Ecosystem Research Station, Huoditang, Ningshan, Shaanxi, China; 3 College of Agriculture, Yangtze University, Jingzhou, Hubei, China; Pacific Northwest National Laboratory, UNITED STATES

## Abstract

As an ecological unit, coarse woody debris (CWD) plays an essential role in productivity, nutrient cycling, carbon sequestration, community regeneration and biodiversity. However, thus far, the information on quantification the decomposition and nutrient content of CWD in forest ecosystems remains considerably limited. In this study, we conducted a long-term (1996–2013) study on decay and nutrient dynamics of CWD for evaluating accurately the ecological value of CWD on the Huoditang Experimental Forest Farm in the Qinling Mountains, China. The results demonstrated that there was a strong correlation between forest biomass and CWD mass. The single exponential decay model well fit the CWD density loss at this site, and as the CWD decomposed, the CWD density decreased significantly. Annual temperature and precipitation were all significantly correlated with the annual mass decay rate. The K contents and the C/N ratio of the CWD decreased as the CWD decayed, but the C, N, P, Ca and Mg contents increased. We observed a significant CWD decay effect on the soil C, N and Mg contents, especially the soil C content. The soil N, P, K, Ca and Mg contents exhibited large fluctuations, but the variation had no obvious regularity and changed with different decay times. The results showed that CWD was a critical component of nutrient cycling in forest ecosystems. Further research is needed to determine the effect of diameter, plant tissue components, secondary wood compounds, and decomposer organisms on the CWD decay rates in the Qinling Mountains, which will be beneficial to clarifying the role of CWD in carbon cycles of forest ecosystems.

## 1. Introduction

Coarse woody debris (CWD) can be produced under conditions of growth competition between trees, natural death of forests at old ages, natural interferences (e.g., wind, rain, snow, fire, lightning, insects, debris flow, and invasion of fungi) and human interferences (logging, hacking trees) [1,2]. As an ecological unit [3], CWD plays an essential role in productivity [4], nutrient cycling [5,6], carbon sequestration [7,8], community regeneration [9] and biodiversity [10,11]. If CWD is not included, it is possible to underestimate global forest detritus by 2–16×10^13^ kg; the relative error associated with this value is 2–10% [12]. Thus, ecologists are paying increasing attention to the ecological functions of CWD in forest ecosystem and its implications for forest management [13].

Until now, the quality and quantity of CWD have been intensively studied in various forest ecosystems around the world, and studies have focused on the ecological role, stocks, respiration and dynamics of CWD [14–19]. However, few quantitative studies have been done on the long term dynamics of the decomposition and nutrient content of CWD in forest ecosystems [20–22]. In China, research on CWD has mainly concerned the concept [23], function [24–26] and stocks of different forest types, such as Korean pine mixed forest [27–30], *Abies fargesii* forest [31], *Castanopsis eyrei* forest [32], evergreen broadleaved forest [33–36], and coniferous forest [37–39]. Studying the influx of CWD may provide an alternative approach for tracking changes in natural forest ecosystems and for predicting the impacts on forests associated with shifts in climate and land use [40]. Due to the high degree of spatial and temporal variability of CWD, it is difficult to get quantitative data on the annual influx of CWD without long term observation of CWD dynamics [41].

The decomposition of CWD is a complex process, including leaching, fragmentation, respiration, and so on [41], which depends on many factors including tree species, temperature, moisture, substrate quality, diameter class, and decomposer type [22,42–45]. It is difficult to measure the decay rate due to the slow nature of the decomposition process and physical fragmentation, which may take decades or even centuries. Using mathematical models to simulate decomposition patterns and estimate the decomposition rate has been widely applied to quantify the decomposition of CWD [41]. Although the single exponential model may not always adequately describe the decay mechanisms as there might be an initial lag until decay starts [46], it is the most common model used to determine the decomposition rates [22,40,42,47,48]. The decay rate can be estimated by relating the time-since-death to the density loss or mass loss of CWD during a given time period [49]. Thus, the most reliable method to determine the decomposition rate is through long term monitoring, which depends on the ability to accurately identify the age of CWD.

In addition, it is thought that CWD releases plentiful carbon, nitrogen, phosphorus and other nutrients during the course of decomposition [42,50]. This enhances the upper forest soil fertility and productivity [4,51], promotes the forest restoration and natural regeneration after harvesting, protects the ecosystem from disturbance-related nutrient losses, and maintains the stability and balance of forest ecosystem [52–54]. However, there are still several questions in the nutrient dynamics of CWD: (1) what is the change pattern of various chemical elements during the decomposition process of CWD? (2) does CWD decay affect the contents of soil chemical elements? (3) what is the released rates of chemical elements from the decomposition of CWD and the accumulated rates of soil chemical elements? To solve these questions, long term studies are needed to quantify the nutrient dynamics during the decomposition process of CWD.

This paper presents the first set of results (1996–2013) from a long-term project measuring CWD at the Qinling National Forest Ecosystem Research Station (QNFERS). *Pinus armandi* and *Quercus aliena* var. *acuteserrata* forests have a considerable wide-ranging distribution over most regions of northern China and constitute a principal zonal forest type in the Qinling Mountains [55]. The Qinling Mountains are an important climate boundary between subtropic and temperate zones in China. The region is distinguished by its high plant and animal diversity, including the last remaining natural habitat of the endangered Giant Panda (*Ailuropoda melanoleuca*) and Japanese Crested Ibis (*Nipponia nippon*) [56].

In this study, we established permanent plots to study CWD dynamics over a 18-year-period. The objectives of this study were to (1) determine the dynamics of CWD mass in permanent plots over a 18-year-period, (2) compare the decay rate estimated using a single exponential decay model and that based on long-term observations of direct measurements of the ratio of mass loss, (3) reveal the change pattern of various chemical elements during the decomposition process of CWD, and (4) evaluate whether CWD decay affects the contents of soil chemical elements.

## 2. Materials and methods

### 2.1. Study area

**Fig 1:**
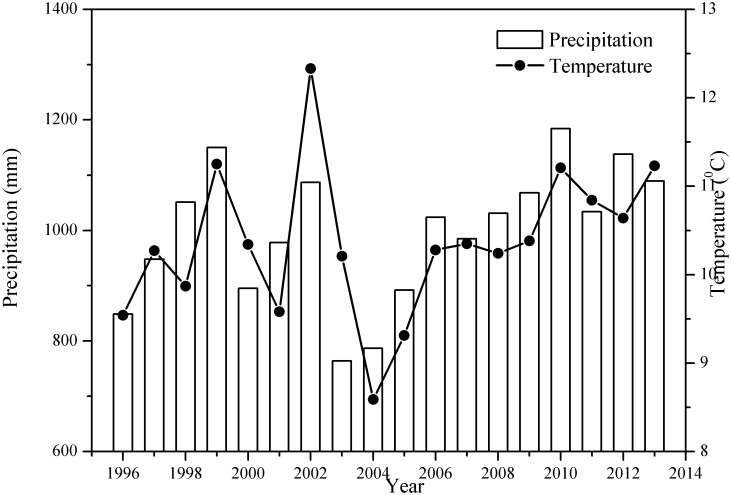
The variation in the mean annual temperature and total precipitation at Huoditang forest region from 1996–2013. The data were sourced from the unpublished Qinling long-term ecological monitoring database.

The study is located on the Huoditang Experimental Forest Farm of Northwest A&F University in the Qinling Mountains, Shaanxi Province, China, and covers an area of 2037 ha. The altitude is 800∼2500 m, the geographic coordinates are N33°18'∼33°28' (latitude) and E108°21'∼108°39' (longitude). The annual average temperature and precipitation during 1996–2013 are shown in Fig 1, which are measured from the weather station (HMP45C, Vaisala, Helsinki, Finland) located 1612 m in this region (N33°26'16″ and E108°26'45″); the climate belongs to the warm temperate zone. The abrupt and broken topography consists mainly of granite and gneiss. The mean slope is 35° and the mean soil depth is 45 cm. The soil units are Cambisols, Umbrisols and Podzols [57]. The study area was selectively logged in the 1960s~70s and since then, there have been no major anthropogenic disturbances except for small amounts of illegal logging. Since the natural forest protection project was initiated in 1998, human activities in this region have largely disappeared.

### 2.2. Ethics statement for field study

The Huoditang forest region is governed by the Huoditang Experimental Tree Farm which is an affiliate of Northwest A&F University. Normally, university stuff can conduct field studies in this place without permissions from the authority. In present study, there were no required specific permissions and endangered or protected species to be included in this field investigation.

### 2.3. Plot measurements

**Fig 2:**
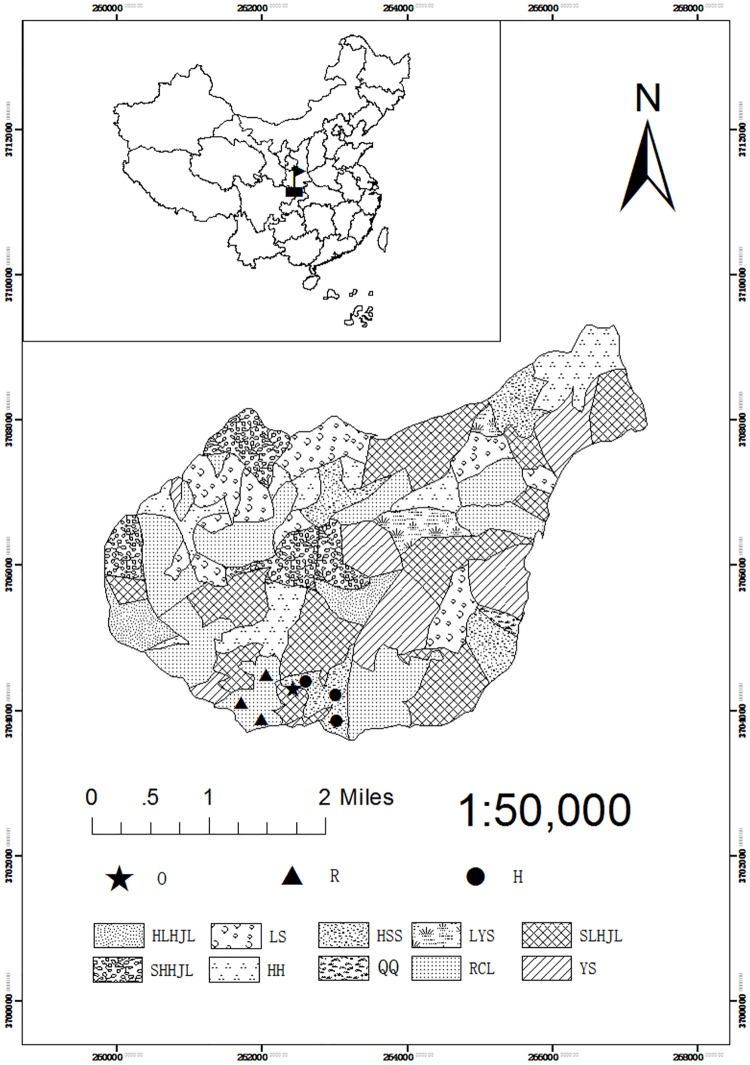
Location of the plots for *P*. *armandi* and *Q*. *aliena* var. *acuteserrata* forests on the Huoditang Experimental Forest Farm in the Qinling Mountains (China). O Weather station; R Plots in *Q*. *aliena* var. *acuteserrata* forest; H Plots in *P*. *armandi* forest; HLHJL Mixed forest between oak and birch; LS *Picea asperata* forest; HSS *P*. *armandi* forest; LYS *Larix principis-rupprechtii* forest; SLHJL Mixed forest between oak and pine; SHHJL Mixed forest between pine and birch; HH *Betula albo-sinensis* forest; QQ *Picea wilsonii* forest; RCL *Q*. *aliena* var. *acuteserrata* forest; YS *Pinus tabulaeformis* forest.

In the summer of 1996, we selected *P*. *armandi* and *Q*. *aliena* var. *acuteserrata* forests for our permanent plots. The plots covered an area of 60 m × 60 m with three replicate plots for *P*. *armandi* and *Q*. *aliena* var. *acuteserrata* forests, and the weather station located 800 m away from the farthest plot (Fig 2). To reduce disturbance, the permanent plots were protected by an enclosure. The three plots were distributed in a nearly flat location with similar site conditions. Each plot was located at least 50 m from the forest edge and was separated from other plots by a buffer strip of at least 20 m. In *P*. *armandi* forest, the altitude is 1524∼1585 m, the geographic coordinates are N33°26'3″∼33°26'29″ and E108°26'51″∼108°27'20″. The *P*. *armandi* forest was 60-years old and was dominated by *P*. *armandi* (85% of trees), with a forest canopy density of 70%. The mean stand height, diameter at breast height (DBH) and stand density were 18 m, 25 cm and 1418 trees・ha^-1^, respectively. In the shrub layer, height varied from 18 cm to 350 cm and the percent cover was 24%. The major shrubs species present were *Euonymus phellomanus*, *Symplocos paniculata*, *Spiraea wilsonii*, *Litsea tsinlingensis* and *Schisandra sphenanthera*, together with herbs, e.g., *Carex leucochlora*, *Lysimachia christinae*, *Rubia cordifolia*, *Houttuynia cordata*, *Pinellia ternata*, *Sedum aizoon*, and ferns. The average height of the herbs was 24 cm and the percent cover was 42%.

For *Q*. *aliena* var. *acuteserrata* forest, the altitude is 1597∼1658 m, the geographic coordinates are N33°26'3″∼33°26'31″ and E108°26'12″∼108°26'38″. The *Q*. *aliena* var. *acuteserrata* forest was 50 years old and was dominated by *Q*. *aliena* var. *acuteserrata* (75% of trees), with a forest canopy density of 80%. The mean stand height, diameter at breast height (DBH) and stand density were 14 m, 20 cm and 1824 trees・ha^-1^, respectively. In the shrub layer, height varied from 64 cm to 560 cm and the percent cover was 18%. The major shrubs species present were *Lonicera hispida*, *Sinarundinaria nitida*, *Symplocos paniculata*, *Lespedeza buergeri* and *Rubus pungens*, together with herbs, e.g., *Spodiopogon sibiricus*, *Epimedium brevicornu*, *Daphne tangutica*, *Urtica fissa*, *Paris quadrifolia*, and *Pteridophyta*. The average height of the herbs was 33 cm and the percent cover was 28%.

We used the USDA Forest Service and Long Term Ecological Research (LTER) definition of CWD (diameter ≥ 10 cm at the widest point) [58]. CWD was categorized in each plot by species, and assigned as logs and snags, as follows [59]: downed or leaning deadwood (> 45° from the vertical) with a minimum diameter ≥ 10 cm at the widest point and length ≥ 1 m, was defined as a log; deadwood ≤ 45° from the vertical with a diameter ≥ 10 cm at the widest point was defined as a snag. When the crown of a tree was withered in August, we considered that this tree was a deadwood and became CWD. Then we documented the date and species, and marked the tree with an aluminum label. We regarded the volume of each piece of log or snag as V_CWD_, and the length and cross-sectional areas at the basal and distal ends of the logs, and the DBH of the snags were documented for each piece of CWD in each plot in August of each year from 1996–2013 to estimate V_CWD_.

If there was no CWD input in our permanent plots in a year, we felled some trees outside the plots to become CWD in these years, and documented the date and species, and marked the felled tree with an aluminum label. These CWD outside the plots were necessary for obtaining each year of decay after the tree had become CWD from 1996–2013. In order to avoid the influence of other factors on the decay, we chose these felled trees outside the plots with similar site conditions as our permanent plots.

### 2.4. Sample preparation

These CWD were allowed to naturally decay, and according to the investigation of the CWD present within and outside the plots from 1996–2013, CWD samples were all collected in August 2013. There were a total of 225 CWD samples (145 within the plots and 80 outside the plots) were collected to calculate the density of the CWD (D_sample_), which included each year of decay after the tree had become CWD from 1996–2013 (Table 1). These samples were collected in their natural state and included bark and vegetation such as mosses growing on the CWD. It should be noted that an assumption of this study is that density was constant throughout a log or snag. When sufficient sound wood was present, the stem and bark of the CWD was cut into disks approximately 2 cm thick using a handsaw. For the more advanced decay classes, the log samples were simply transferred onto aluminum plates. The samples were immediately sealed in plastic bags, transported to the laboratory, and the sample volume (V_sample_) was determined gravimetrically by water displacement. The CWD samples were then dried to a constant weight at 70°C. The D_sample_ was estimated as the ratio of dry mass to V_sample_.

**Table 1:** The numbers of CWD samples at various decay times in the *P*. *armandi* and *Q*. *aliena* var. *acuteserrata* forests.

Distribution zone	Forest types	Decay times (year)																	Total
		1	2	3	4	5	6	7	8	9	10	11	12	13	14	15	16	17	
**Plots**	***P*. *armandi***	7		7		5	6		9	5	8		7		6	6		6	72
	***Q*. *aliena* var. *acuteserrata***	8		7	9		8		7		9			6	7	6		6	73
**Outside the plots**	***P*. *armandi***		6		6			7				5		8			6		38
	***Q*. *aliena* var. *acuteserrata***		7			6		6		5		6	7				5		42

### 2.5. Estimation of the decay rate

Decomposition can be expressed as either density or mass loss. In our study, decay rates are estimated from the measured density of CWD. A single exponential decay model is used to determine the decay rate (k), which is the most commonly used and accepted decay model [22,40,42,47,48].

$${\text{y}}_{\text{t}}={\text{y}}_{0}{\text{e}}^{-\text{kt}}$$

where y_t_ is the density of the CWD at time t, y_0_ is the initial density of the CWD, and t is the decay time.

The time to lose 50% and 95% of the density was estimated from the decay rate:

$${\text{T}}_{0.5}=-\text{ln}\left(0.5\right)/\text{k}=0.693/\text{k}$$

$${\text{T}}_{0.95}=-\text{ln}\left(0.05\right)/\text{k}=2.996/\text{k}$$

For comparison with the single exponential decay model, another method based on long-term observations to calculate the mass decay rate (k') was used:

$$\text{k}\prime ={\text{D}}_{\text{CWD}}/{\text{B}}_{\text{CWD}}$$

$${\text{D}}_{\text{CWD}}={\text{I}}_{\text{CWD}}-\Delta_{\text{CWD}}$$

where D_CWD_ is the mass of decomposed CWD over a given time period; I_CWD_ is the input of CWD mass (when a tree became CWD, we calculated the new CWD mass per year as I_CWD_); Δ_CWD_ is the increment of CWD mass (from one year to the next), which implies the net change from one CWD census to the next, and would therefore include CWD input and decomposition; B_CWD_ is the CWD mass.

### 2.6. Calculation of forest biomass

Forest biomass includes the mass of living trees, shrubs, herbs, litter and CWD. The species type and DBH of all living trees in each plot were documented in August of each year from 1996–2013 to estimate (the annual change in) the biomass, which was calculated using a regression model developed in a previous study in this region (Table 2) [60]. Five 2 m × 2 m shrub, and 1 m × 1 m nested herbal subplots were also established in the four corners and the middle of each plot in August of each year from 1996–2013 to estimate the biomass of the shrubs, herbs and litter. The aboveground biomass of the shrubs, herbs and litter was quantified by harvesting, and the belowground biomass of the shrubs and herbs was quantified by digging. A pit (50 cm × 50 cm) was dug for the biomass of the coarse and fine roots in each shrub, and the belowground organs (roots, rhizomes, tubers) of herb species were thoroughly excavated in these subplots.

**Table 2:** The regression model of biomass, volume and height in the *P*. *armandi* and *Q*. *aliena* var. *acuteserrata* forests.

Forest types	Contents	Regression equation	Correlation coefficient	Reliability of 95% of the estimated accuracy
***Q*. *aliena* var. *acuteserrata***	**Stem**	ln*W*_*s*_ = 0.99253 ln(*D*^2^ *H*) − 3.78818	0.99763	94.24
	**Bark**	ln*W*_*BA*_ = 0.75632 ln(*D*^2^ *H*) − 3.92450	0.99708	95.37
	**Branch**	ln*W*_*B*_ = 3.49934 ln*D* − 6.50726	0.96524	84.27
	**Leaf**	ln*W*_*L*_ = 2.29344 ln*D* − 4.88581	0.97832	84.45
	**Root**	ln*W*_*R*_ = 2.76435 ln*D* − 4.20817	0.99106	89.15
	**Stem volume**	ln*V*_*s*_ = 0.96884ln(*D*^2^ *H*) − 10.07352	0.99807	96.85
	**Bark volume**	ln*V*_*BA*_ = 0.65531ln(*D*^2^ *H*) − 9.43191	0.99392	94.99
	**Height**	$\frac{1}{\text{H}}=\frac{8.01921}{{\text{D}}^{2.59222}}+0.05263$	0.78814	95.60
***P*. *armandi***	**Stem**	ln*W*_*S*_ = 1.02363 ln(*D*^2^ *H*) − 4.49970	0.99802	97.09
	**Bark**	ln*W*_*BA*_ = 0.88417 ln(*D*^2^ *H*) − 5.38472	0.99698	96.73
	**Branch**	ln*W*_*B*_ = 2.57551 ln*D* − 4.08452	0.98656	90.60
	**Leaf**	ln*W*_*L*_ = 2.75687 ln*D* − 5.75891	0.98004	81.56
	**Root**	ln*W*_*R*_ = 0.97120 ln(*D*^2^ *H*) − 5.26301	0.97927	92.13
	**Stem volume**	ln*V*_*s*_ = 0.95697ln(*D*^2^ *H*) − 9.95783	0.99843	96.27
	**Bark volume**	ln*V*_*BA*_ = 0.78772ln(*D*^2^ *H*) − 10.48352	0.99771	97.09
	**Height**	$\frac{1}{\text{H}}=\frac{1.34537}{{\text{D}}^{1.70800}}+0.07143$	0.88076	98.52

Note: D Diameter at breast height (cm); H Height of tree (m); W_S_ Dry weight of stem (kg); W_BA_ Dry weight of bark (kg); W_B_ Dry weight of branch (kg); W_L_ Dry weight of leaf (kg); W_R_ Dry weight of roots (kg); V_S_ Stem volume (m^3^); V_BA_ Bark volume (m^3^).

Prior to calculating the CWD mass, Smalian’s formula was used to calculate the volume of each log sample based on the length and cross-sectional areas at the basal and distal ends of an assumed cylinder [61].

$$\text{V}=\text{L}\left[\frac{\pi {\left({\text{D}}_{1}/2\right)}^{2}+\pi {\left({\text{D}}_{2}/2\right)}^{2}}{2}\right]$$

where L (m) is the length of the piece of log, and D is the diameter (m), at either end. It should be noted that this formula tends to slightly overestimate volume due to the natural taper of the material [62]. For snags, we inserted the height and diameter of each relevant sample into a species-specific wood volume equation (Table 2) [60].

Finally, the CWD mass (t·ha^-1^) was calculated as the product of D_sample_ and V_CWD_.

### 2.7. Chemical analyses

To compare the effect of CWD species on the chemical elements, we chose these CWD samples presenting the same decay year between *P*. *armandi* and *Q*. *aliena* var. *acuteserrata* CWD. There were a total of 113 CWD samples collected from our permanent plots in August 2013 (Table 1). All samples were collected in their natural state and included bark and vegetation such as mosses growing on the CWD. When sufficient sound wood was present, the stem and bark of the CWD was cut into disks, approximately 5 cm thick, using a handsaw. For the more advanced decay classes, the CWD samples were simply transferred onto aluminum plates. Soil samples (included litter layer) were chose from three soil layers (0–10 cm, 10–20 cm, 20–40 cm) underneath the collected CWD samples in the *P*. *armandi* and *Q*. *aliena* var. *acuteserrata* forests. 16 soil profiles (2 soil profiles in each decay year) with a distance of >5 m from each other were selected in each plot. A total of 96 (16 × 3 × 2) soil profiles (60 cm × 40 cm) were excavated in August 2013. In each plot, each 2 samples from the same decay year and soil layer were pooled into one composite sample (approximately 500 g). All samples were immediately sealed in plastic bags and transported to the laboratory.

The CWD samples were dried in an oven at 70°C for 72 hours, and the soil samples were air dried at room temperature for 2 weeks. All of the dried samples were ground to pass through a 100-mesh screen for chemical analyses. Total carbon (C) was determined using potassium dichromate oxidation titration; total nitrogen (N) and phosphorus (P) were measured using the Kjeldahl nitrogen method and Mo anti-antimony colorimetry, respectively; the potassium (K) content was measured using a flare photometer and the calcium (Ca) and magnesium (Mg) contents were determined using atomic absorption spectrometry.

### 2.8. Statistical analyses

One-way ANOVA with SAS 8.0 software were used to determine the effect of the CWD tree species on the CWD mass; the effects of the mean annual temperature and total annual precipitation on the CWD decay rate; the effect of the decay time, soil depth, and CWD species on the chemical elements; the effect of the decay time on the CWD density; and the effect of the soil depth and CWD species on the average annual contents of the chemical elements. The mean decay rates for the two methods were also compared using ANOVA with SAS 8.0 software. If there were significant effects, Duncan’s t-test was used to compare the differences. Pearson’s correlation coefficients (r) were calculated to test the dependence of the CWD mass on the forest biomass using SAS 8.0 software.

According to our long term investigation, a total of 145 densities of the CWD samples with different decay times were calculated in *P*. *armandi* and *Q*. *aliena* var. *acuteserrata* plots (Table 1). A single exponential decay model was used to simulate the relationship between these densities and decay times. The model was fitted using exponential regression with Origin 8.0 software, and the *P* value was employed for the fitting of the model. The goodness of fit was evaluated based on R^2^. Decay rates were estimated separately for each *P*. *armandi* and *Q*. *aliena* var. *acuteserrata* plot.

Based on 18 years observations, we calculated the 51 annual mass decay rates (k') separately for *P*. *armandi* and *Q*. *aliena* var. *acuteserrata* CWD. Meanwhile, the mean annual temperatures and total annual precipitations were measured from the weather station during the 18 years. The relationship between mean annual temperatures and the annual mass decay rates of the corresponding year was fitted using exponential regression with Origin 8.0 software, and the *P* value was employed for the fitting of the model. The goodness of fit was evaluated based on R^2^. Pearson’s correlation coefficient (r) was calculated to test the dependence of the annual precipitation on the annual mass decay rates of the corresponding year using SAS 8.0 software.

A map of the location was made using the ArcGIS 10.2.2 software. All calculations and statistical analyses used the plot as the experimental unit (N = 3), with a *P* value of 0.05 set as the limit for statistical significance.

## 3. Results

### 3.1. Dynamics of the CWD mass

**Fig 3:**
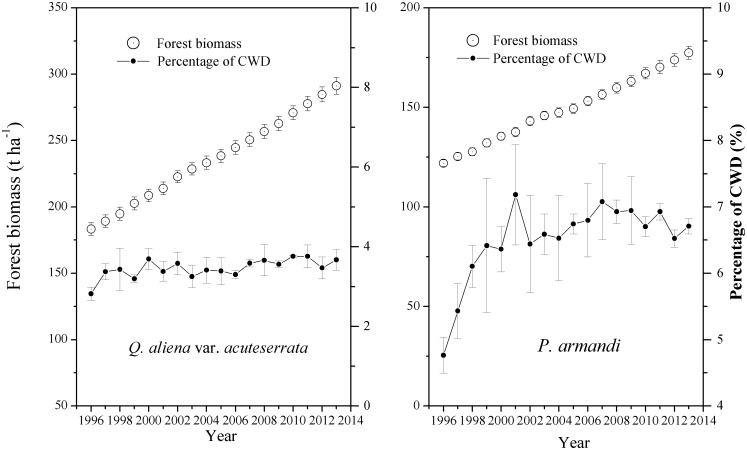
The forest biomass and the percentage of CWD to the forest biomass (the mass of living trees, shrubs, herbs, litter and CWD) in the *P*. *armandi* and *Q*. *aliena* var. *acuteserrata* forests from 1997–2013. The forest biomass data sources are Chen and Peng [60] and the unpublished Qinling long-term ecological monitoring database. Errors bars are based on plot as experimental unit (N = 3).

**Fig 4:**
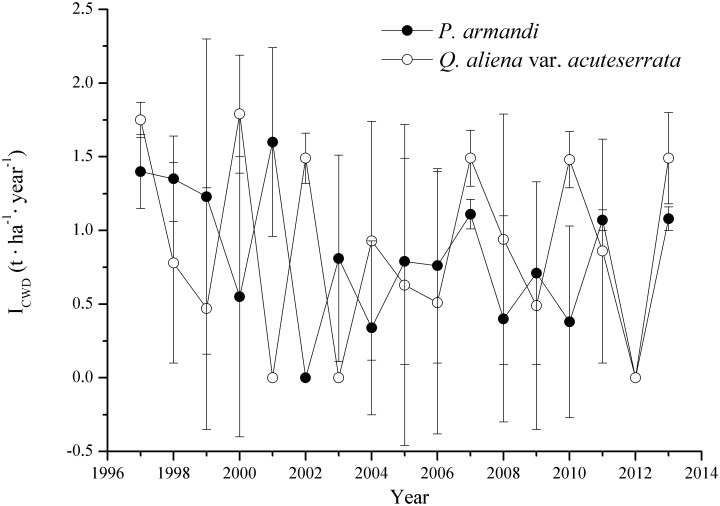
The variation in annual I_CWD_ in the *P*. *armandi* and *Q*. *aliena* var. *acuteserrata* forests from 1997–2013. I_CWD_ is the input of CWD mass (when a tree became CWD, we calculated the new CWD mass per year as I_CWD_). Errors bars are based on plot as experimental unit (N = 3).

The average annual B_CWD_ in the *P*. *armandi* forest (9.78±1.82 t·ha^-1^) was significantly higher than that in the *Q*. *aliena* var. *acuteserrata* forest (8.23±1.63 t·ha^-1^) (*P*<0.0001) from 1996–2013 (Fig 3), but there was no significant difference between average annual I_CWD_ in the *P*. *armandi* forest (0.80±0.67 t·ha^-1^) and that in the *Q*. *aliena* var. *acuteserrata* forest (0.89±0.78 t·ha^-1^) (Fig 4, *P* = 0.53). There were obvious variations in the annual I_CWD_ in the *P*. *armandi* and *Q*. *aliena* var. *acuteserrata* forests, but there was no obvious regularity. The average annual forest biomass in the *Q*. *aliena* var. *acuteserrata* forest (218.28±34.45 t·ha^-1^) was significantly higher than that in the *P*. *armandi* forest (149.24±16.91 t·ha^-1^) (*P*<0.0001), and the annual forest biomass of both forests increased linearly from 1996–2013. The average annual biomass increment in the *Q*. *aliena* var. *acuteserrata* forest (6.34±1.07 t·ha^-1^) was significantly higher than that in the *P*. *armandi* forest (3.27±0.99 t·ha^-1^) (*P*<0.0001), but the average percentage of CWD in the *P*. *armandi* forest (6.51%±0.70%) was significantly higher than that in the *Q*. *aliena* var. *acuteserrata* forest (3.46%±0.32%) (*P*<0.0001). The B_CWD_ increased significantly in the *P*. *armandi* and *Q*. *aliena* var. *acuteserrata* forests with an increase in the forest biomass (*P*<0.0001). There was a strong correlation between the forest biomass and the B_CWD_; the *Q*. *aliena* var. *acuteserrata* forest exhibited a slightly stronger correlation (r = 0.92) compared with the *P*. *armandi* forest (r = 0.90).

### 3.2. CWD decay rate

**Fig 5:**
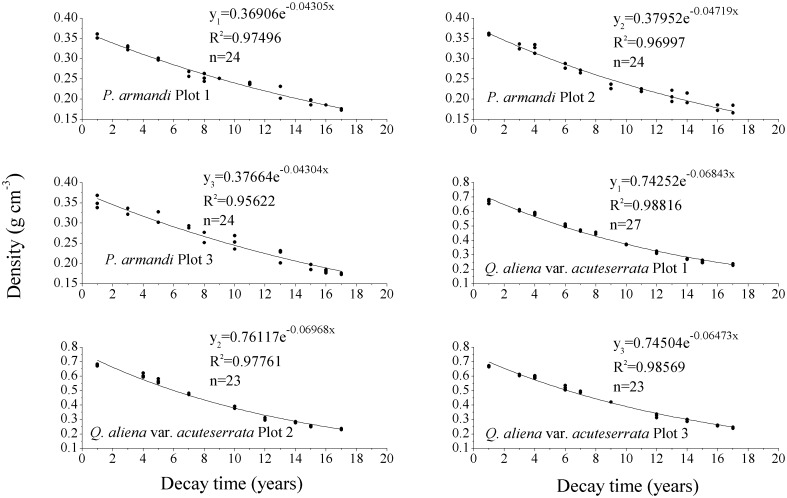
Decomposition of CWD in the *P*. *armandi* and Q. *aliena* var. *acuteserrata* plots. The relationship between density and decomposition was simulated using a single exponential decay model.

**Fig 6:**
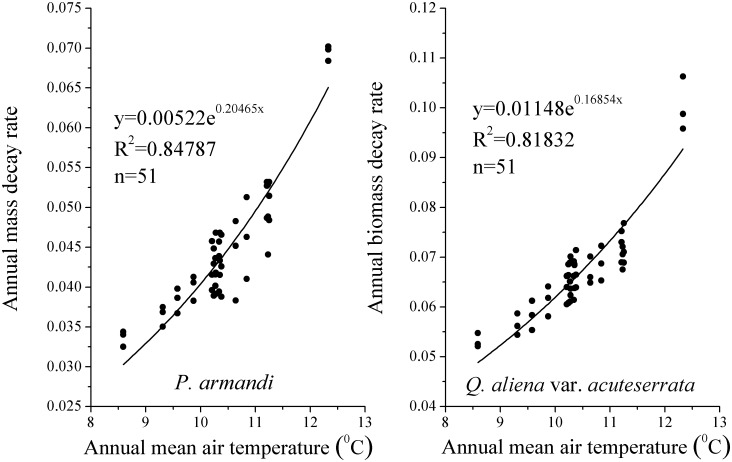
The relationship between the annual mass decay rate and annual temperature in the *P*. *armandi* and *Q*. *aliena* var. *acuteserrata* forests from 1997–2013.

The CWD density of *Q*. *aliena* var. *acuteserrata* was significantly higher than that of *P*. *armandi* at different decay times (Fig 5, *P*<0.0001); the CWD density decreased significantly with the decomposition of *Q*. *aliena* var. *acuteserrata* and *P*. *armandi* (*P*<0.0001). The relationship between CWD density and the decay time was simulated using a single exponential decay model, and the average decay rate (k) of *P*. *armandi* CWD was 0.04±0.002 a^-1^ (R^2^ = 0.97±0.01), while that of *Q*. *aliena* var. *acuteserrata* CWD was 0.07±0.003 a^-1^ (R^2^ = 0.98±0.006). The single exponential decay model predicted that it would take 16 and 67 years to decompose 50% and 95% of *P*. *armandi* CWD, compared with 10 and 44 years to decompose 50% and 95%, respectively of *Q*. *aliena* var. *acuteserrata* CWD. Based on long-term observations, we calculated the average annual D_CWD_ of *P*. *armandi* forest, i.e., 0.44±0.08 t·ha^-1^, while that of the *Q*. *aliena* var. *acuteserrata* forest was 0.56±0.09 t·ha^-1^ from 1997–2013. The average annual mass decay rate (k') of *Q*. *aliena* var. *acuteserrata* CWD (0.07±0.02 a^-1^) was significantly higher than that of *P*. *armandi* CWD (0.05±0.01 a^-1^) from 1997–2013 (Fig 6, *P*<0.0001). There was no significant difference between the two methods with respect to the estimated decay rate of *P*. *armandi* CWD (*P* = 0.33) and *Q*. *aliena* var. *acuteserrata* CWD (*P* = 0.13). With an increase in annual temperature, the annual k' of *P*. *armandi* and *Q*. *aliena* var. *acuteserrata* CWD increased significantly (*P*<0.0001), and annual temperature was strongly exponentially correlated with annual k'. Moreover, there was a significant correlation between the annual precipitation and the annual k' (*P*<0.0001, r = 0.53).

### 3.3. Contents of chemical elements and the C/N ratio of the CWD

**Fig 7:**
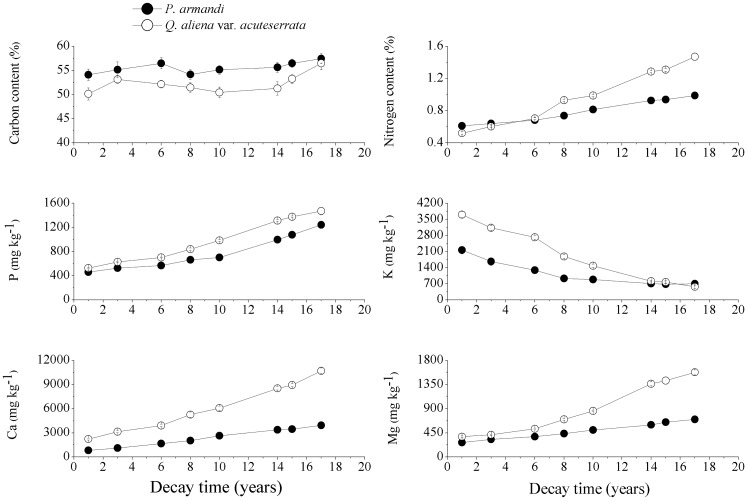
The contents of the chemical elements in the *P*. *armandi* and *Q*. *aliena* var. *acuteserrata* CWD at different decay times. Errors bars are based on plot as experimental unit (N = 3).

The contents of 6 chemical elements (C, N, P, K, Ca, Mg) in *P*. *armandi* and *Q*. *aliena* var. *acuteserrata* CWD differed over time; C was present in the highest amount, followed by N (Fig 7, *P*<0.0001). The K contents decreased as the *P*. *armandi* and *Q*. *aliena* var. *acuteserrata* CWD decomposed, but the contents of C, N, P, Ca and Mg increased. Compared with the *P*. *armandi* CWD, the contents of the 6 chemical elements in the *Q*. *aliena* var. *acuteserrata* CWD exhibited more significant variation as the CWD decayed. The average annual K contents (mg·g^-1^·a^-1^) released from the decomposition of the *Q*. *aliena* var. *acuteserrata* CWD (0.20±0.06) were significantly higher than those from the *P*. *armandi* CWD (0.09±0.04), and the average annual C, N, P, Ca and Mg contents (mg·g^-1^·a^-1^) resulting from the decomposition of the *Q*. *aliena* var. *acuteserrata* CWD (3.98±0.76, 0.60±0.17, 0.06±0.004, 0.52±0.13 and 0.08±0.03) were also significantly higher than those from the *P*. *armandi* CWD (2.09±0.44, 0.24±0.06, 0.05±0.007, 0.20±0.04 and 0.03±0.006, respectively) (*P*<0.05).

**Fig 8:**
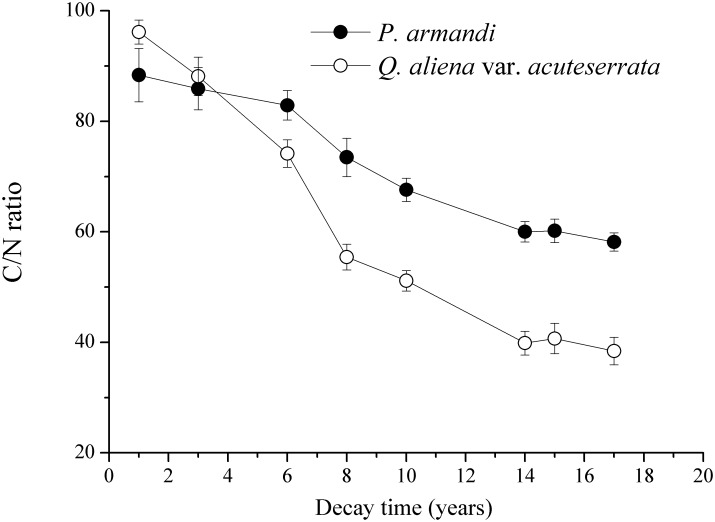
The C/N ratio in the *P*. *armandi* and *Q*. *aliena* var. *acuteserrata* CWD at different decay times. Errors bars are based on plot as experimental unit (N = 3).

As the CWD of *P*. *armandi* and *Q*. *aliena* var. *acuteserrata* decomposed, the C/N ratio decreased significantly, and the C/N ratio of the *Q*. *aliena* var. *acuteserrata* CWD exhibited more significant variation than that of the *P*. *armandi* CWD (Fig 8, *P*<0.01). However, during the 14 and 17 years of decay in the *P*. *armandi* and *Q*. *aliena* var. *acuteserrata* CWD, the decrease of the C/N ratio tended to get slower. There was no significant difference in the C/N ratio between the *P*. *armandi* (85.88±3.14) and *Q*. *aliena* var. *acuteserrata* CWD (88.11±3.48) after 3 years of decay (*P* = 0.12). However, the C/N ratio of the *Q*. *aliena* var. *acuteserrata* CWD (96.13±2.15) was significantly higher than that of the *P*. *armandi* CWD (88.34±4.85) after 1 year of decay (*P*<0.05), and the C/N ratio of the *P*. *armandi* CWD was significantly higher than that of the *Q*. *aliena* var. *acuteserrata* CWD at the other decay times (*P*<0.05).

### 3.4. Contents of soil chemical elements under CWD

**Fig 9:**
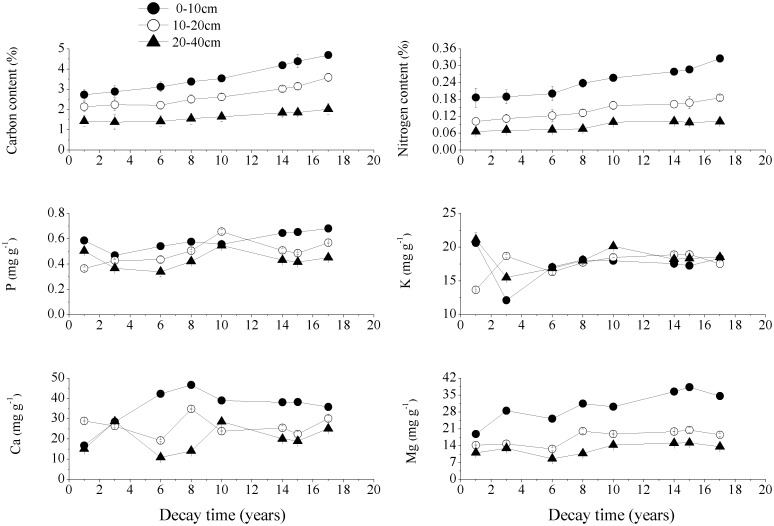
The contents of the soil chemical elements in the three soil layers under the *P*. *armandi* CWD at different decay times. Errors bars are based on plot as experimental unit (N = 3).

**Fig 10:**
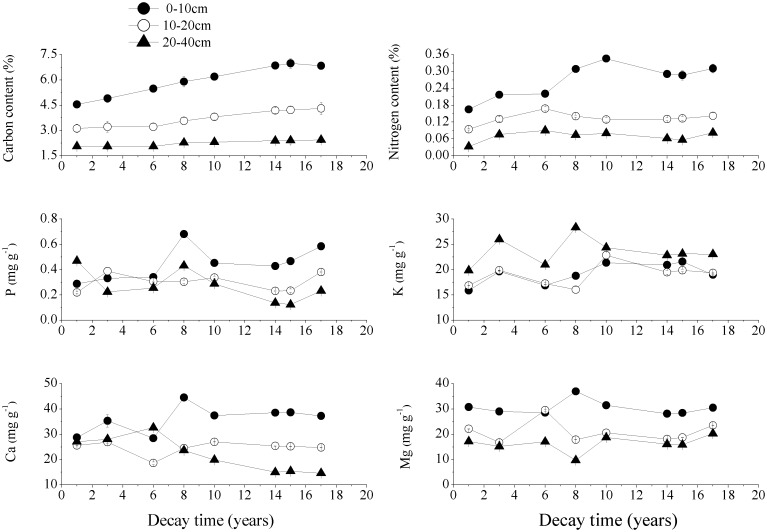
The contents of the soil chemical elements in the three soil layers under the *Q*. *aliena* var. *acuteserrata* CWD at different decay times. Errors bars are based on plot as experimental unit (N = 3).

As the soil depth increased, the soil C and N contents under the *P*. *armandi* and *Q*. *aliena* var. *acuteserrata* CWD decreased significantly at all decay times, while the soil Mg content under the *P*. *armandi* CWD was similar (Figs 9 and 10, *P*<0.05). As the CWD decomposed, the C and N contents in the three soil layers under the *P*. *armandi* CWD and the soil C content under the *Q*. *aliena* var. *acuteserrata* CWD increased. In the 0–10 cm soil layer in particular, these element contents exhibited significantly higher accumulation as the CWD decayed. As the CWD decomposed, there were significant fluctuations in the P, K, Ca and Mg contents measured in the three soil layers under the *P*. *armandi* CWD and in the soil N, P, K, Ca, Mg contents under the *Q*. *aliena* var. *acuteserrata* CWD, but there was no obvious regularity, and the fluctuations varied with different decay times.

With an increase in soil depth, there were significant (*P*<0.05) decreases in the average annual soil C and N contents under the *P*. *armandi* and *Q*. *aliena* var. *acuteserrata* CWD, the average annual soil P and Ca contents under the *Q*. *aliena* var. *acuteserrata* CWD, and the average annual soil Mg content under the *P*. *armandi* CWD. No significant differences were observed in the contents of the other elements (Table 3, *P*>0.05). The average annual Mg content in the 10–20 cm soil layer and the average annual Ca and P contents in the 20–40 cm soil layer under the *Q*. *aliena* var. *acuteserrata* CWD, and the average annual soil K content in the 10–20 and 20–40 cm soil layers under the *P*. *armandi* CWD were negative, whereas the other values were positive. Positive numbers indicate that as the CWD decomposed, the contents of the soil chemical elements increased. However, most of the negative values did not appear to be significantly different from 0, which implies that the contents of the soil chemical elements did not change significantly as the CWD decomposed. There were significant differences between the *P*. *armandi* and *Q*. *aliena* var. *acuteserrata* CWD with respect to the average annual N content in the 10–20 cm soil layer, the average annual Ca content in the 20–40 cm soil layer, and the average annual Mg content in the 0–10 cm soil layer (*P*<0.05), but no significant difference was observed for the remaining elements (*P*>0.05). Moreover, in the 0–10 cm and 10–20 cm soil layers, the average annual soil C content under the *Q*. *aliena* var. *acuteserrata* CWD was significantly higher than the other soil chemical elements (*P*<0.05). In the 20–40 cm soil layer, the average annual Ca content under the *Q*. *aliena* var. *acuteserrata* CWD was significantly lower than the other soil chemical elements (*P*<0.05). Under the *P*. *armandi* CWD, the average annual soil C, Ca and Mg contents were significantly higher than the average annual soil N, P and K contents in the 0–10 cm layer, and the average annual soil C content was significantly higher than the other soil chemical elements in the 10–20 cm layer (*P*<0.05), but there was no significant difference between the contents of the average annual soil chemical elements in the 20–40 cm soil layer (*P*>0.05).

**Table 3:** The contents of the average annual soil chemical elements accumulated under the *P*. *armandi* and *Q*. *aliena* var. *acuteserrata* CWD.

CWD tree species	Soil layers	Average annual accumulation of soil chemical elements (mg·g^-1^·a^-1^)					
		C	N	P	K	Ca	Mg
***P*. *armandi***	**0–10 cm**	1.25 a (0.33)	0.09 a (0.04)	0.008 a (0.02)	-0.006 a (0.96)	0.84 a (2.26)	0.97 a (1.25)
	**10–20 cm**	0.91 b (0.48)	0.05 b (0.02)	0.01 a (0.02)	0.21 a (0.58)	0.02 a (2.30)	0.40 b (0.86)
	**20–40 cm**	0.39 c (0.21)	0.02 c (0.03)	0.002 a (0.03)	-0.007 a (0.69)	0.59 a (2.64)	0.26 b (0.66)
***Q*. *aliena* var. *acuteserrata***	**0–10 cm**	1.50 a (0.60)	0.09 a (0.13)	0.02 a (0.05)	0.25 a (0.64)	0.56 a (1.99)	0.02 a (1.17)
	**10–20 cm**	0.81 b (0.37)	0.01 b (0.07)	0.005 ab (0.03)	0.22 a (0.85)	0.10 a (0.99)	-0.05 a (1.75)
	**20–40 cm**	0.28 c (0.23)	0.01 b (0.06)	-0.01 b (0.04)	0.14 a (1.12)	-1.00 b (1.18)	0.19 a (1.34)

Note: Means within a column followed by different letters are significantly different at *P*<0.05; the standard errors are provided in parentheses, are based on plot as experimental unit (N = 3).

## 4. Discussion

### 4.1. Dynamics of CWD mass

The B_CWD_ of the *P*. *armandi* and *Q*. *aliena* var. *acuteserrata* forests in the Qinling Mountains was at the lower limit of global records (8–200 t·ha^-1^) [12] and was lower than that measured in the monsoon evergreen broad-leaved forest of Dinghushan (38.54 t·ha^-1^) [40] and an *Abies fargesii* forest close to our study site (15.85 t·ha^-1^) [31]. The comparatively low B_CWD_ may be caused by a lower I_CWD_; the average annual I_CWD_ at this site was lower than that measured in a monsoon evergreen broad-leaved forest of Dinghushan (1.32 t·ha^-1^) [40] and in an *Abies fargesii* forest close to our study site (1.88 t·ha^-1^) [31]. The lower I_CWD_ cannot be explained by major anthropogenic and natural disturbances in these forests, except for a combination of strong winds and steep topography, diseases and pests. The B_CWD_ of the *P*. *armandi* forest was mainly caused by *Dendroctonus armandi* infestation. This explanation might reveal why the B_CWD_ in the *P*. *armandi* forest was significantly higher than that in the *Q*. *aliena* var. *acuteserrata* forest. In addition, the methods used to estimate B_CWD_ may be another reason for the lower B_CWD_. For example, one methodological difference between studies is whether they exclude or include standing dead trees. Additionally, plot size, or transect length, may influence whether the sampling adequately captures the full spatial variation in forest structure [62].

Our results showed that the annual increment and biomass of the broad-leaved forest were significant higher than those of the coniferous forest, and the biomass of both forest types increased each year. In our study, there was a strong correlation between B_CWD_ and forest biomass. This result has been reported in several studies [63–65]. However, the dependence of B_CWD_ on forest biomass differs between forest types. The *Q*. *aliena* var. *acuteserrata* forest showed a slightly stronger correlation compared with the *P*. *armandi* forest because it had experienced more constant mortality in the past [66]. Moreover, the small diameter classes, primary decomposition stages, single species composition (data not shown) and lower proportion of B_CWD_ to forest biomass in these forests compared with the 10% reported for boreal old-growth forests [67] may illustrate the strong correlation between B_CWD_ and forest biomass. The disturbance history of the study area included selective logging in the 1960s~70s and the establishment of the permanent plots in 1996, which was also a crucial factor for the dependence of B_CWD_ on forest biomass [66].

### 4.2. CWD decay rate

In our study, the changes in CWD density had been used to simulate the decomposition of CWD. Density was used by many studies of CWD decay [17,40,48,68,69], but few researchers concluded that the mass was a more reliable parameter [46,70]. Both mass and density change as wood decays, and generally follow a negative exponential pattern. However, mass is dependent on both the density and volume of the CWD; therefore, once CWD volume depletion begins, mass loss follows a decay trajectory that differs from that of density [71]. Density sometimes decreases progressively until advanced decay classes are apparent and then stabilizes, but mass, or volume, does not stabilize, and even continues to decline [72,73]. Consequently, density loss may not describe the actual decay correctly, especially during advanced decay [70]. However, our chronosequence approach covered only 17 years of the decay process, and during this period it was appropriate and convenient to simulate CWD decomposition this way. Longer-term studies are certainly necessary for the mass loss to obtain more accurate estimation of decay rates in future.

The CWD decay rate in our study was higher than that in the Changbai Mountain (*Pinus koraiensis*, 0.0162 a^-1^) [12], but lower than that in Dinghushan (*Schima superba*, 0.1486 a^-1^) [40]. Air temperature is probably the main reason for this difference; the annual average temperature in this region (10.4°C) was higher than that in the Changbai Mountain (3.9°C) [12], but lower than that in Dinghushan (21.5°C) [40]. After 18 years of observation, we found a strong exponential correlation between air temperature and the CWD decay rate. Within a suitable temperature range, as the temperature rose, the activation of microorganism accelerated, and the CWD decay rates increased exponentially. This conclusion has been reported in several studies [42,45,74]. An analysis of a global dataset on CWD decay rates showed that the annual average temperature is a main driver of decomposition, accounting for 34% of the variation in decay rates [45]. However, in the Russian southern boreal forest, Yatskov et al. did not find correlation between decomposition rates and temperature [75]. In addition, our study concluded that there was a positive correlation between the annual precipitation and the CWD decay rate, which was similar to other studies [7,76,77]. Although with the increase of moisture content, the activation of microorganism accelerated and the CWD decay rates increased, the growth of microorganism would be inhibited in an anaerobic environment with too much water [19].

In our study, the two CWD tree species had different decay rates; the slower decomposition of the coniferous tree species compared with the broad-leaved tree species was consistent with Zhang et al. [78]. Substrate quality and decomposer organisms may explain this phenomenon [5,17,43,79–81]. Coniferous trees have a simple structure, little leaf organization, and more resistant compounds such as tannins, resins, waxes, and polyphenols; whereas broad-leaved trees have more sugar, amylum and protein, which are easily decomposed [43,80,82]. For example, due to existing polyphenols, *Pseudotsuga menziesii* had a lower decay rate (0.005–0.010 a^-1^) compared with other tree species [12]. Our study showed that the average annual N and P that accumulated from the decomposition of the *Q*. *aliena* var. *acuteserrata* CWD were significantly higher than those from the *P*. *armandi* CWD, which is another reason for the higher decay rate of the *Q*. *aliena* var. *acuteserrata* CWD. High nutrient contents, especially N and P contents in CWD, can provide better conditions for the activities of microbes and other invertebrates [43,80].

We estimated the CWD decay rate only for stem wood and bark; little is known about the decomposition of other CWD parts such as roots and branches [5,45,83]. Moreover, there are various viewpoints on the relationship between CWD diameter and the decay rate [22,42,84]. Therefore, more research is needed to determine the effect of diameter, plant tissue components, secondary wood compounds, and decomposer organisms on the CWD decay rates in the Qinling Mountains, which will be beneficial to provide a scientific basis to clarify the role of CWD in carbon cycles of forest ecosystems.

### 4.3. Changes in chemical elements during decomposition of CWD

C is the most important element in CWD, and thus its decomposition makes a considerable contribution to the loss of CWD mass. As the CWD decayed, the C content increased slightly, which was similar with some studies [49,85–87]. A small increase in C content was probably linked to the loss of polysaccharides and the relative increase in lignin over time [88], and indicated that C loss was generally slightly slower than mass loss. Moreover, the increase in C content had implications for the calculation of C pool changes of CWD over time [20].

However, some studies have suggested that C content does not change significantly during decomposition [5,89,90]. They suggested that C loss was equal to mass loss during the CWD decays. In addition, a part of the C in the CWD is decomposed by microorganisms and is released to the atmosphere in the form of CO_2_; other parts are damaged by leaching and fragmentation in the soil [41]. As the CWD decomposed, the N accumulated significantly, which may have been caused by nitrogen fixation by fungi and nitrogen inputs from bulk precipitation [40,70,89,91,92]. The C/N ratio decreased significantly with increasing decay, and CWD with a high C/N ratio is more difficult to decompose [20,40,86,93]. P is resistant to leaching, and as the CWD decomposed, the P accumulated significantly, which may be due to a lower leaching rate compared with the loss of CWD mass [84,89,94]. The K content in the bark is higher than that in sapwood and heartwood [5,40,88,95]. Therefore, the decrease in the K content was significantly correlated with the decomposition of bark, and the faster leaching rate compared with mass loss may be another reason for the significant decrease in the K content. The significant increases in the Ca and Mg contents may be caused by 1) a lower leaching rate of Ca and Mg compared with the loss of CWD mass, and 2) increasing numbers of microbes and mosses associated with the CWD decay. In particular, during advanced decay, there was 90% coverage of the CWD with mosses and tracheophytes, which contained high Ca and Mg contents.

### 4.4. Contents of soil chemical elements under CWD

CWD decay had a significant effect on the contents of the soil chemical elements, especially the soil C content, which is consistent with Ge et al. [96]. However, other researchers have reported relatively insignificant effects [6,70,97,98]. Plant residues are the main components of soil, which is formed primarily by the decomposition of CWD; thus, the soil C content increased significantly as the CWD decayed. The soil N, P, K, Ca and Mg contents exhibited large fluctuations, which may be due to environmental factors that affected the decomposition of the CWD. Moreover, substances leached from the CWD affected the chemical properties and enzyme activity in soil, which changed the soil nutrient contents. As the CWD decomposed, the variation in the N, P, K, Ca and Mg contents indicated no obvious regularity, but we considered that the CWD plays an important role in the case of a poor soil condition or after disturbance [12,41,99].

In our study, we did not measure the contents of the soil chemical elements without CWD, which was lacking an appropriate control. However, these soil samples were all taken in August 2013 from a chronosequence of decaying CWD going back 17 years, which may reduce the background variation in the contents of the soil chemical elements, and also may minimize the impact on lacking an appropriate control. By analyzing and comparing the contents of the soil chemical elements, we believed that there was a significant variation at the different CWD decay times. If CWD was removed, the contents of the soil chemical elements would decrease (especially in C and N contents), and may inhibit the nutrient cycling and reduce the forest productivity, ultimately could restrict the stability of forest ecosystems. Our research revealed the change pattern of various nutrients during the decomposition process of CWD, which may be beneficial for establishing proper management practices and promoting the nutrient cycling and regeneration of forest ecosystems.

## 5. Conclusion

Our study provided the first set of results (1996–2013) from a long-term project measuring CWD in northwestern China. Using permanent plots, we revealed the dynamics of CWD mass over 18 years, which showed a strong correlation between forest biomass and CWD mass. CWD is important for eco-forestry, but the amount and characteristics of CWD to be retained need further research. Development of CWD reasonable strategies was indispensable for future forest management. By comparing the decay rate, we found that a single exponential model could be used to simulate the decomposition of CWD tree species in this region. Annual temperature and precipitation were all significantly correlated with the annual mass decay rate. These results will allow forest managers to better understand the status of CWD decomposition. We revealed the change pattern of various chemical elements during the decomposition process of CWD, and concluded that the effect of CWD decay on the contents of soil chemical elements was significant, especially the soil C content. This shows that CWD is a critical component of nutrient cycling in forest ecosystems. The results from our study will be helpful for the measurement process involved in improving the nutrient cycling and forest regeneration of forest ecosystems and also for facilitating the conversion of fragile forests into productive and healthy forest ecosystems in the future.

## Supporting information

S1 Table Dynamics of CWD biomass (t·ha^-1^) and decay rate (k') in the *P*. *armandi* and *Q*. *aliena* var. *acuteserrata* forests during 1996–2013. (DOCX)

S2 Table Some photos of plots and weather station in this region. (DOCX)
