# Supplementary material for: Decay and nutrient dynamics of coarse woody debris in the Qinling Mountains, China
Source: PLoS One. 2017 Apr 6;12(4):e0175203. doi: 10.1371/journal.pone.0175203 (PMC5383274; doi:10.1371/journal.pone.0175203)
Supplement: S1 Table — (DOCX) [file pone.0175203.s001.docx]

S1 Table. Dynamics of CWD biomass (t·ha^-1^) and decay rate (k’) in the *P. armandi* and *Q. aliena* var. *acuteserrata* forests during 1996-2013

| Sample plots | Items | Year | | | | | | | | | | | | | | | | | |
| --- | --- | --- | --- | --- | --- | --- | --- | --- | --- | --- | --- | --- | --- | --- | --- | --- | --- | --- | --- |
|  |  | 1996 | 1997 | 1998 | 1999 | 2000 | 2001 | 2002 | 2003 | 2004 | 2005 | 2006 | 2007 | 2008 | 2009 | 2010 | 2011 | 2012 | 2013 |
| *P. armandi* 1# | B_CWD_ | 5.37 | 6.23 | 7.53 | 8.86 | 8.45 | 9.35 | 8.70 | 9.54 | 9.23 | 9.93 | 10.62 | 11.21 | 10.80 | 11.43 | 10.92 | 11.46 | 11.00 | 11.65 |
|  | I_CWD_ |  | 1.24 | 1.64 | 1.88 | 0 | 1.22 | 0 | 1.26 | 0 | 1.04 | 1.12 | 1.05 | 0 | 1.08 | 0 | 1.02 | 0 | 1.17 |
|  | Δ_CWD_ |  | 0.86 | 1.30 | 1.33 | -0.41 | 0.90 | -0.65 | 0.84 | -0.31 | 0.70 | 0.69 | 0.59 | -0.41 | 0.63 | -0.51 | 0.54 | -0.46 | 0.65 |
|  | D_CWD_ |  | 0.38 | 0.34 | 0.55 | 0.41 | 0.32 | 0.65 | 0.42 | 0.31 | 0.34 | 0.43 | 0.46 | 0.41 | 0.45 | 0.51 | 0.48 | 0.46 | 0.52 |
|  | k’ |  | 0.0708 | 0.0546 | 0.0730 | 0.0463 | 0.0379 | 0.0695 | 0.0483 | 0.0325 | 0.0368 | 0.0433 | 0.0433 | 0.0366 | 0.0417 | 0.0446 | 0.0440 | 0.0401 | 0.0473 |
| *P. armandi* 2# | B_CWD_ | 5.89 | 6.75 | 7.47 | 6.95 | 8.16 | 9.06 | 8.38 | 9.11 | 8.80 | 9.81 | 9.40 | 10.02 | 10.80 | 10.34 | 10.98 | 11.65 | 11.21 | 11.75 |
|  | I_CWD_ |  | 1.27 | 1.07 | 0 | 1.64 | 1.25 | 0 | 1.16 | 0 | 1.34 | 0 | 1.06 | 1.21 | 0 | 1.13 | 1.15 | 0 | 1.03 |
|  | Δ_CWD_ |  | 0.86 | 0.72 | -0.52 | 1.21 | 0.90 | -0.68 | 0.73 | -0.31 | 1.01 | -0.41 | 0.62 | 0.78 | -0.46 | 0.64 | 0.67 | -0.44 | 0.54 |
|  | D_CWD_ |  | 0.41 | 0.35 | 0.52 | 0.43 | 0.35 | 0.68 | 0.43 | 0.31 | 0.33 | 0.41 | 0.44 | 0.43 | 0.46 | 0.49 | 0.48 | 0.44 | 0.49 |
|  | k’ |  | 0.0696 | 0.0519 | 0.0696 | 0.0619 | 0.0429 | 0.0751 | 0.0513 | 0.0340 | 0.0375 | 0.0418 | 0.0468 | 0.0429 | 0.0426 | 0.0474 | 0.0437 | 0.0378 | 0.0437 |
| *P. armandi* 3# | B_CWD_ | 6.17 | 7.46 | 8.38 | 9.64 | 9.26 | 11.26 | 10.60 | 10.18 | 10.85 | 10.47 | 11.22 | 12.01 | 11.60 | 12.21 | 11.70 | 12.27 | 11.80 | 12.31 |
|  | I_CWD_ |  | 1.68 | 1.34 | 1.81 | 0 | 2.34 | 0 | 0 | 1.02 | 0 | 1.16 | 1.23 | 0 | 1.06 | 0 | 1.05 | 0 | 1.03 |
|  | Δ_CWD_ |  | 1.29 | 0.92 | 1.26 | -0.38 | 2.00 | -0.66 | -0.42 | 0.67 | -0.38 | 0.75 | 0.79 | -0.41 | 0.61 | -0.51 | 0.57 | -0.47 | 0.51 |
|  | D_CWD_ |  | 0.39 | 0.42 | 0.55 | 0.38 | 0.34 | 0.66 | 0.42 | 0.35 | 0.38 | 0.41 | 0.44 | 0.41 | 0.45 | 0.51 | 0.48 | 0.47 | 0.52 |
|  | k’ |  | 0.0632 | 0.0563 | 0.0656 | 0.0394 | 0.0367 | 0.0586 | 0.0396 | 0.0344 | 0.0350 | 0.0392 | 0.0392 | 0.0341 | 0.0388 | 0.0418 | 0.0410 | 0.0383 | 0.0441 |
| *Q. aliena* var. *acuteserrata* 1# | B_CWD_ | 4.84 | 5.96 | 5.48 | 6.24 | 7.08 | 6.63 | 7.23 | 6.68 | 7.63 | 7.14 | 8.17 | 8.89 | 10.03 | 9.52 | 10.15 | 10.72 | 10.19 | 10.66 |
|  | I_CWD_ |  | 1.64 | 0 | 1.42 | 1.38 | 0 | 1.34 | 0 | 1.37 | 0 | 1.54 | 1.28 | 1.67 | 0 | 1.27 | 1.14 | 0 | 1.15 |
|  | Δ_CWD_ |  | 1.12 | -0.48 | 0.76 | 0.84 | -0.45 | 0.60 | -0.55 | 0.95 | -0.49 | 1.03 | 0.72 | 1.14 | -0.51 | 0.63 | 0.57 | -0.53 | 0.47 |
|  | D_CWD_ |  | 0.52 | 0.48 | 0.66 | 0.54 | 0.45 | 0.74 | 0.55 | 0.42 | 0.49 | 0.51 | 0.56 | 0.53 | 0.51 | 0.64 | 0.57 | 0.53 | 0.68 |
|  | k’ |  | 0.1074 | 0.0850 | 0.1204 | 0.0865 | 0.0636 | 0.1116 | 0.0761 | 0.0629 | 0.0642 | 0.0714 | 0.0685 | 0.0596 | 0.0509 | 0.0672 | 0.0562 | 0.0494 | 0.0667 |
| *Q. aliena* var. *acuteserrata* 2# | B_CWD_ | 5.12 | 6.45 | 7.11 | 6.43 | 7.70 | 7.23 | 8.13 | 7.61 | 8.64 | 8.17 | 7.65 | 8.63 | 8.12 | 9.04 | 10.00 | 9.41 | 8.85 | 9.74 |
|  | I_CWD_ |  | 1.87 | 1.12 | 0 | 1.82 | 0 | 1.67 | 0 | 1.43 | 0 | 0 | 1.54 | 0 | 1.46 | 1.62 | 0 | 0 | 1.58 |
|  | Δ_CWD_ |  | 1.33 | 0.66 | -0.68 | 1.27 | -0.47 | 0.90 | -0.52 | 1.03 | -0.47 | -0.52 | 0.98 | -0.51 | 0.92 | 0.96 | -0.59 | -0.56 | 0.89 |
|  | D_CWD_ |  | 0.54 | 0.46 | 0.68 | 0.55 | 0.47 | 0.77 | 0.52 | 0.40 | 0.47 | 0.52 | 0.56 | 0.51 | 0.54 | 0.66 | 0.59 | 0.56 | 0.69 |
|  | k’ |  | 0.1055 | 0.0713 | 0.0956 | 0.0855 | 0.0610 | 0.1065 | 0.0640 | 0.0526 | 0.0544 | 0.0637 | 0.0732 | 0.0591 | 0.0665 | 0.0730 | 0.0590 | 0.0595 | 0.0780 |
| *Q. aliena* var. *acuteserrata* 3# | B_CWD_ | 5.54 | 6.74 | 7.44 | 6.73 | 8.32 | 7.83 | 8.53 | 8.00 | 7.56 | 8.94 | 8.40 | 9.46 | 10.07 | 9.51 | 10.39 | 11.22 | 10.59 | 11.66 |
|  | I_CWD_ |  | 1.75 | 1.22 | 0 | 2.17 | 0 | 1.47 | 0 | 0 | 1.88 | 0 | 1.64 | 1.15 | 0 | 1.56 | 1.44 | 0 | 1.75 |
|  | Δ_CWD_ |  | 1.2 | 0.7 | -0.71 | 1.59 | -0.49 | 0.70 | -0.53 | -0.44 | 1.38 | -0.54 | 1.06 | 0.61 | -0.56 | 0.88 | 0.83 | -0.63 | 1.07 |
|  | D_CWD_ |  | 0.55 | 0.52 | 0.71 | 0.58 | 0.49 | 0.77 | 0.53 | 0.44 | 0.50 | 0.54 | 0.58 | 0.54 | 0.56 | 0.68 | 0.61 | 0.63 | 0.68 |
|  | k’ |  | 0.0993 | 0.0771 | 0.0954 | 0.0862 | 0.0589 | 0.0983 | 0.0621 | 0.0550 | 0.0661 | 0.0604 | 0.0691 | 0.0571 | 0.0556 | 0.0715 | 0.0587 | 0.0562 | 0.0642 |

Note: D_CWD_ is the mass of decomposed CWD over a given time period (D_CWD_= I_CWD_-Δ_CWD_); I_CWD_ is the input of CWD mass (when a tree became CWD, we calculated the new CWD mass per year as I_CWD_); Δ_CWD_ is the increment of CWD mass (from one year to the next), which implies the net change from one CWD census to the next, and would therefore include CWD input and decomposition; B_CWD_ is the CWD mass; k’ is the mass decay rate based on long-term observations (k’= D_CWD_ /B_CWD_).
