# Supplementary figures and images for: Decay and nutrient dynamics of coarse woody debris in the Qinling Mountains, China
Source: PLoS One. 2017 Apr 6;12(4):e0175203. doi: 10.1371/journal.pone.0175203 (PMC5383274; doi:10.1371/journal.pone.0175203)

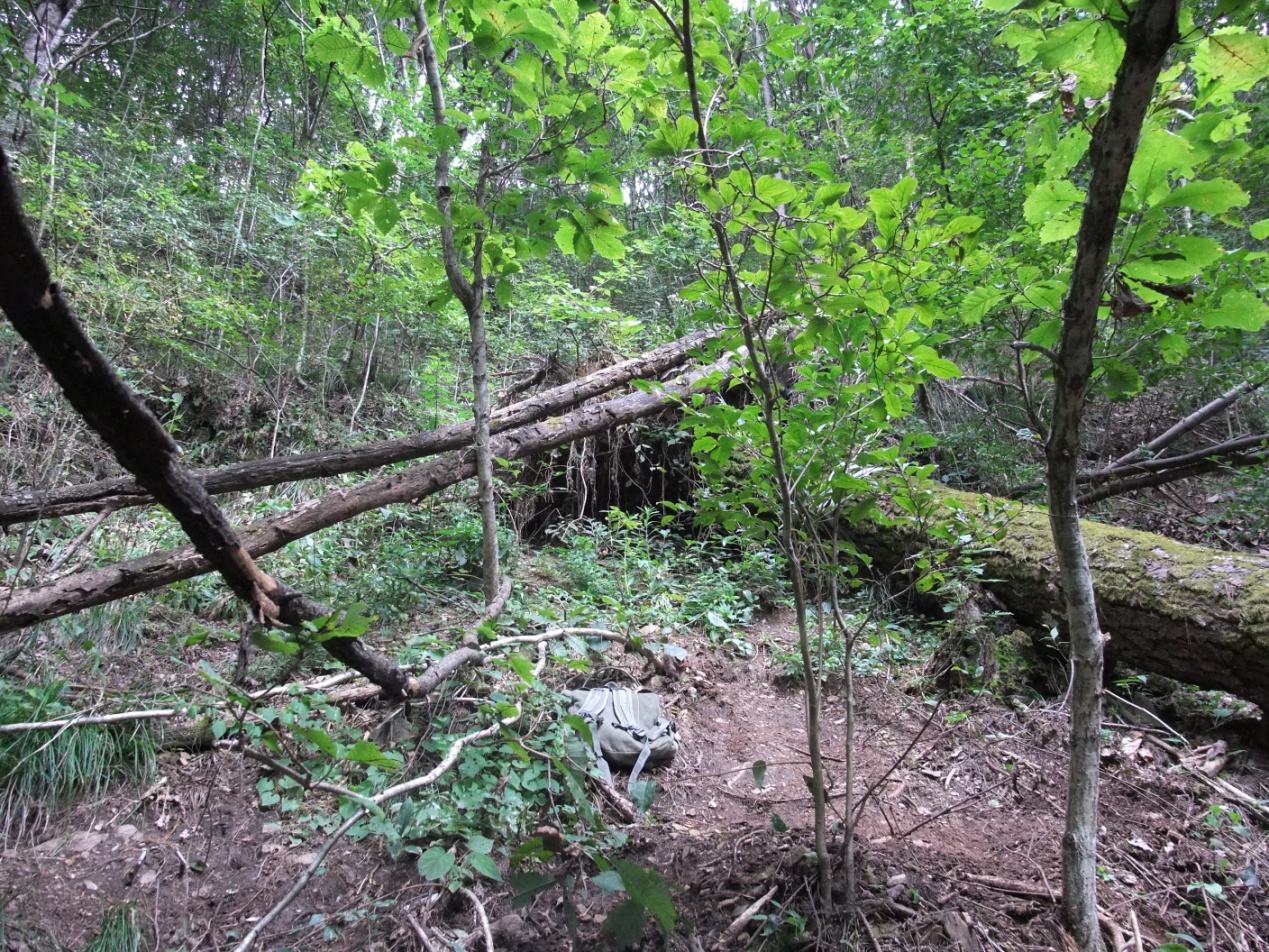

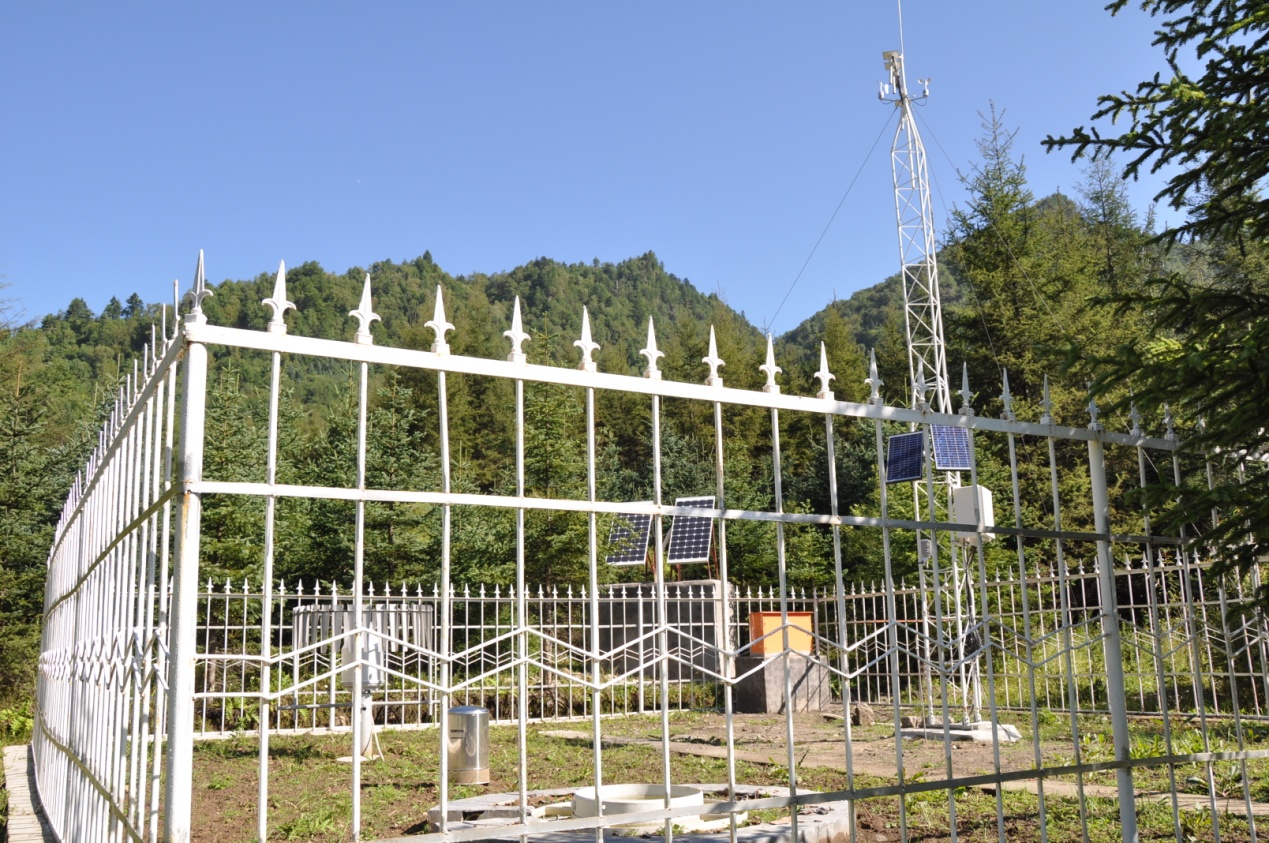

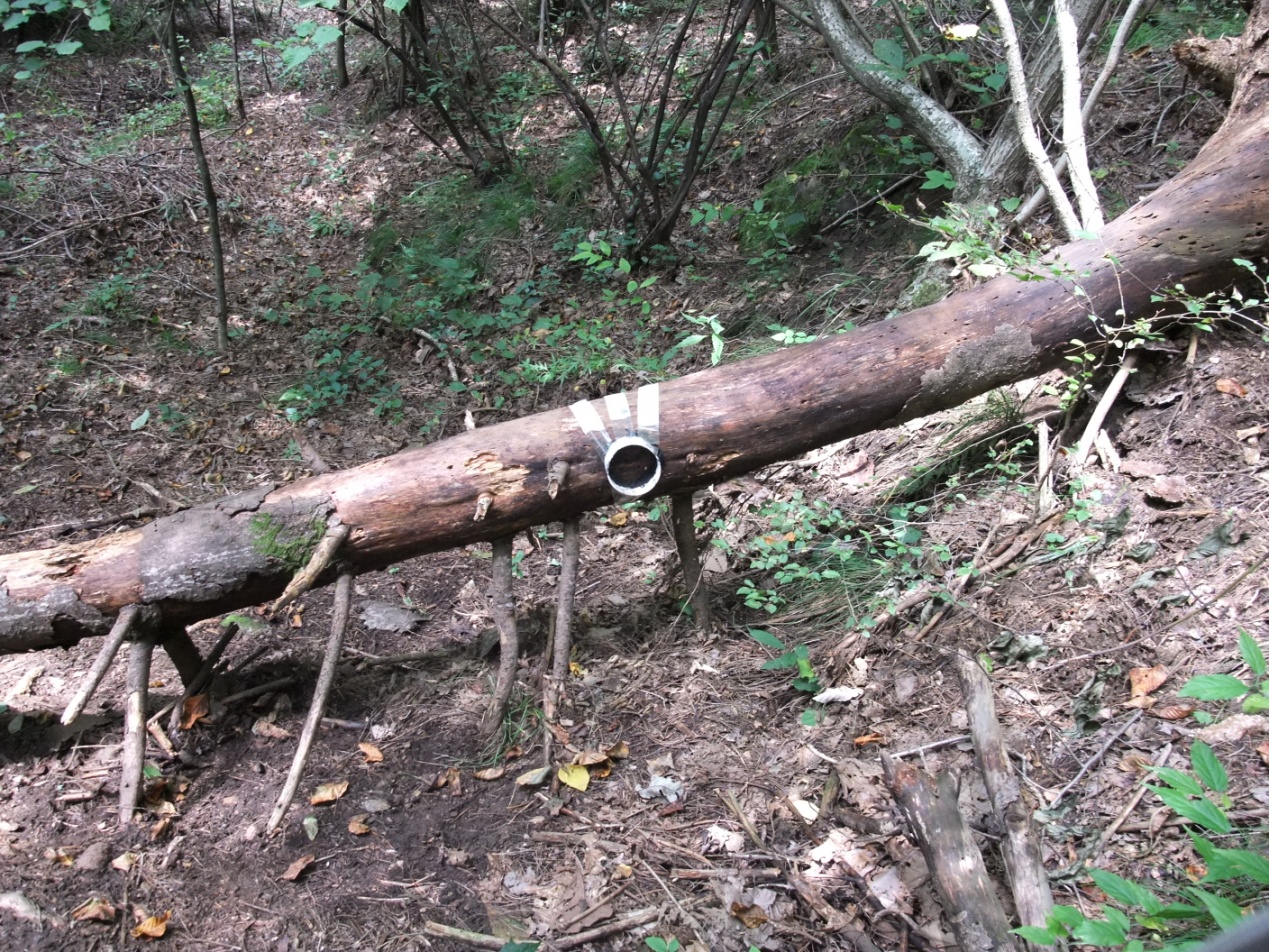

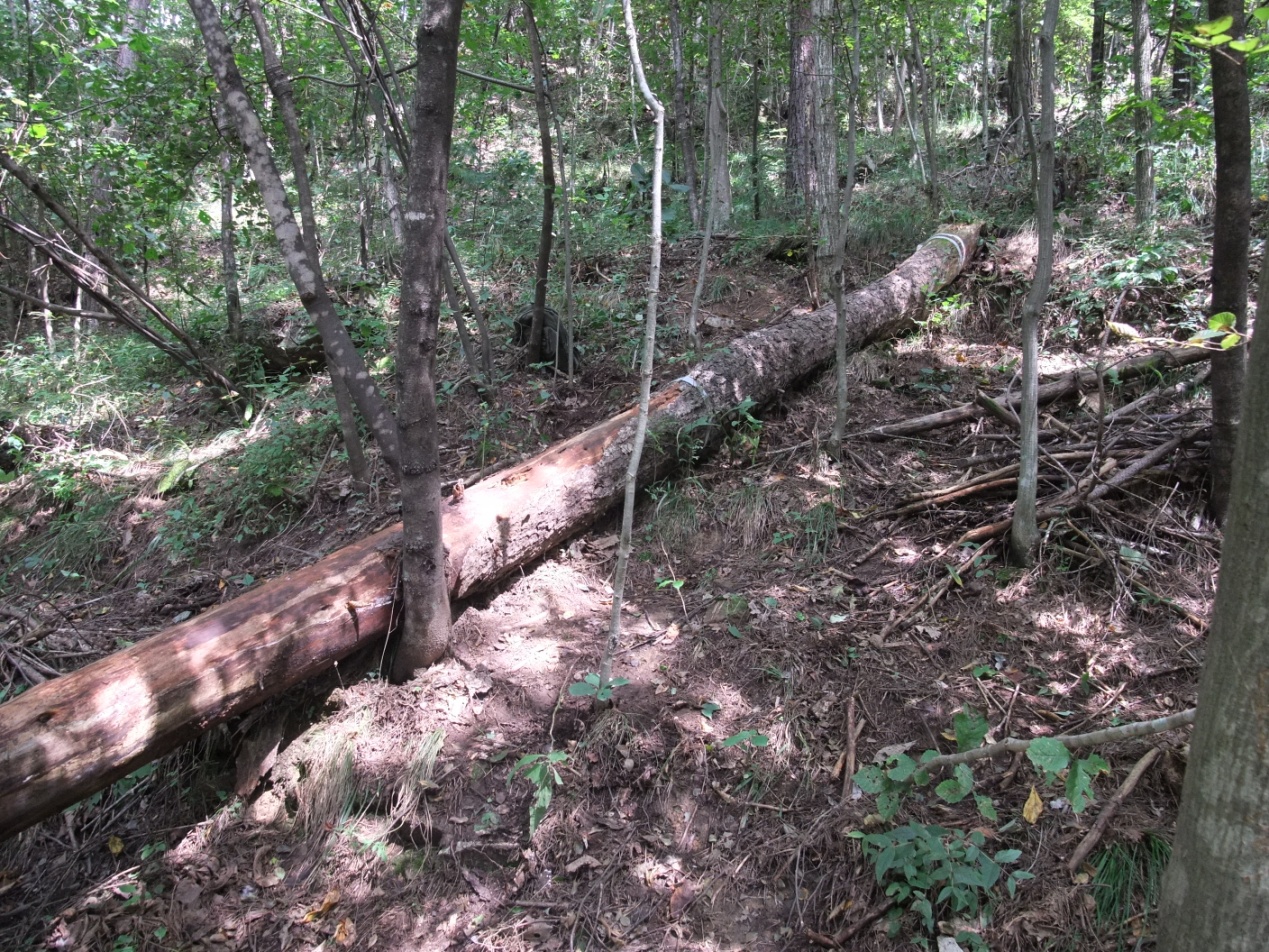

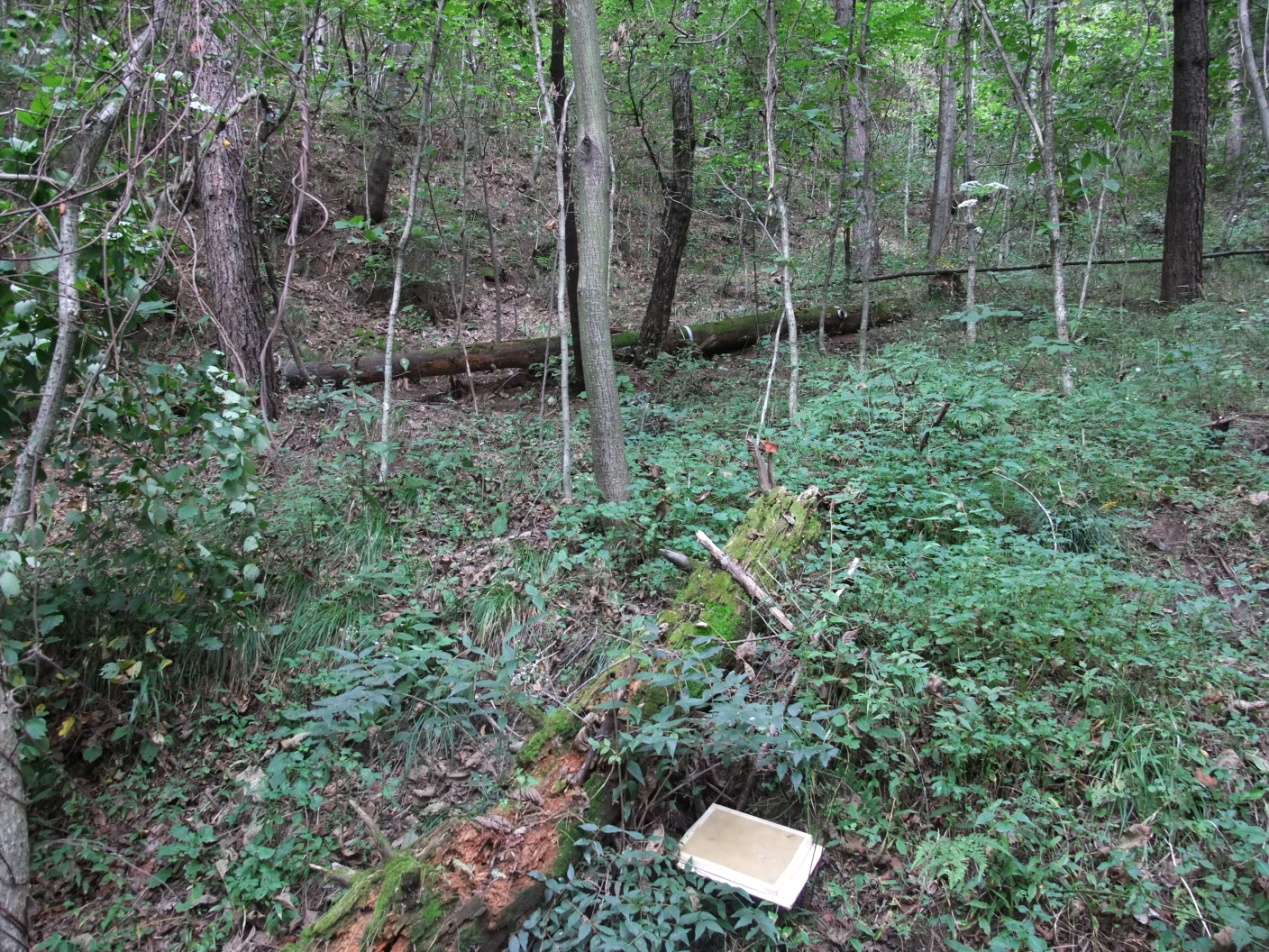

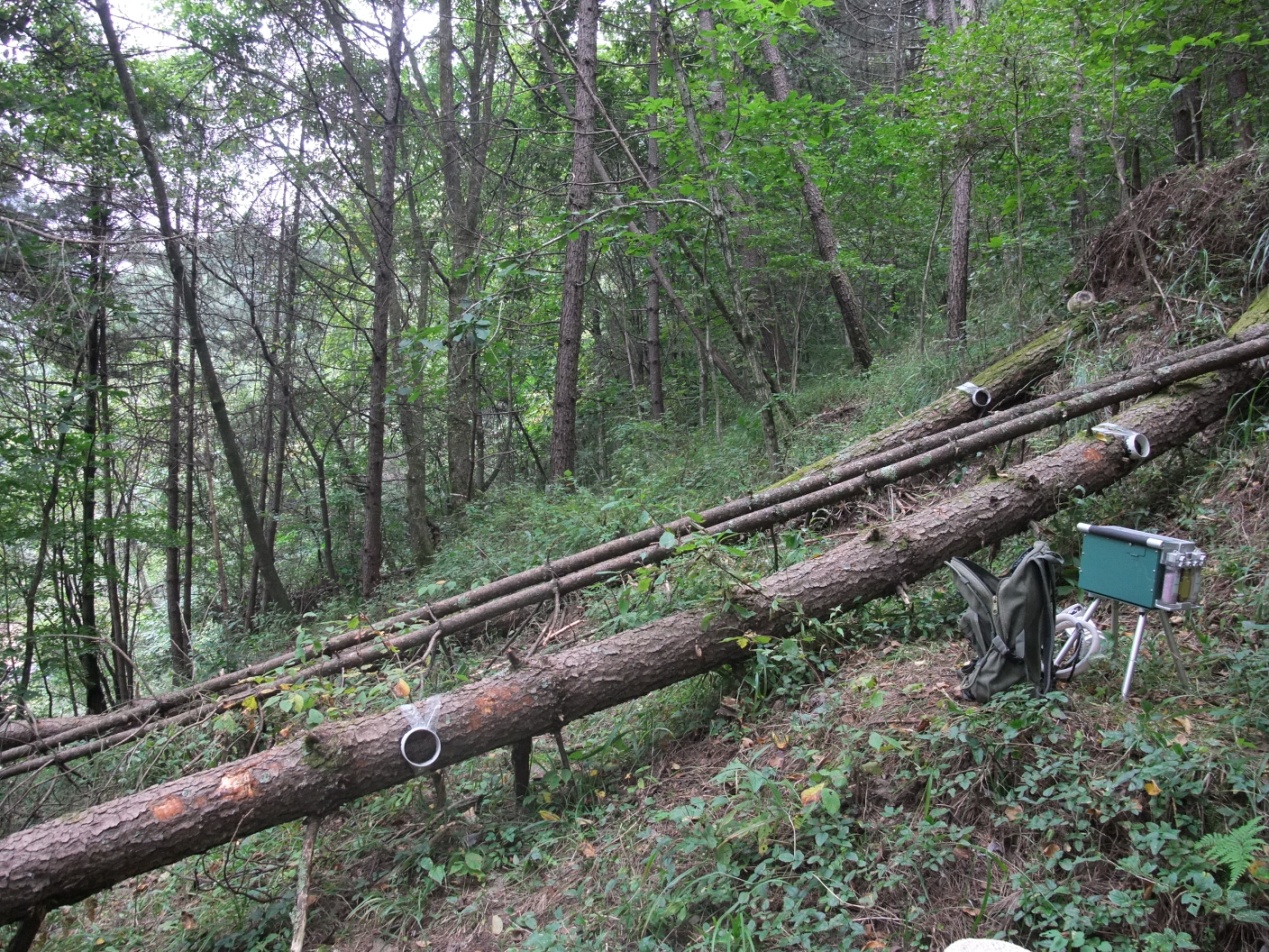

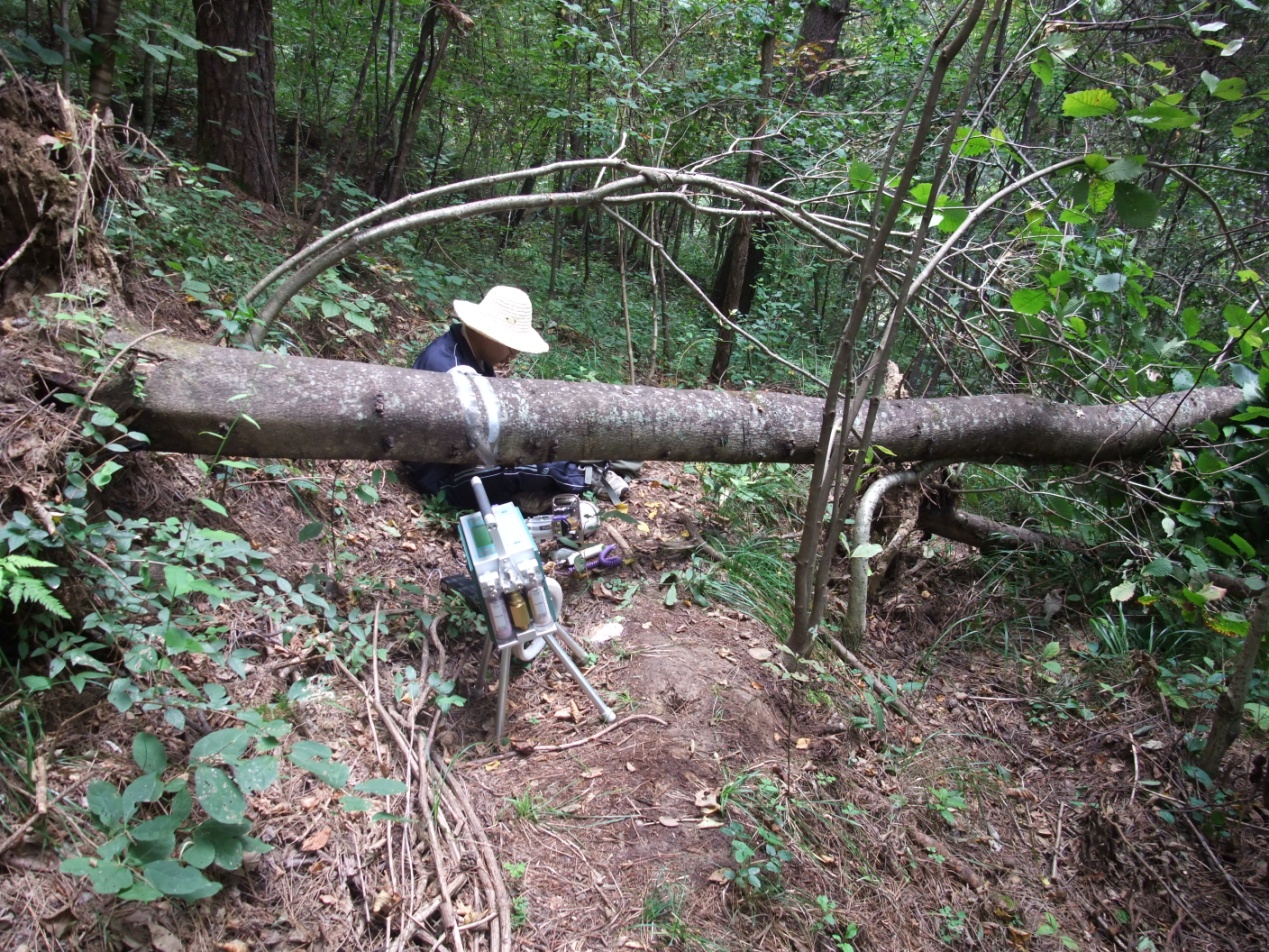

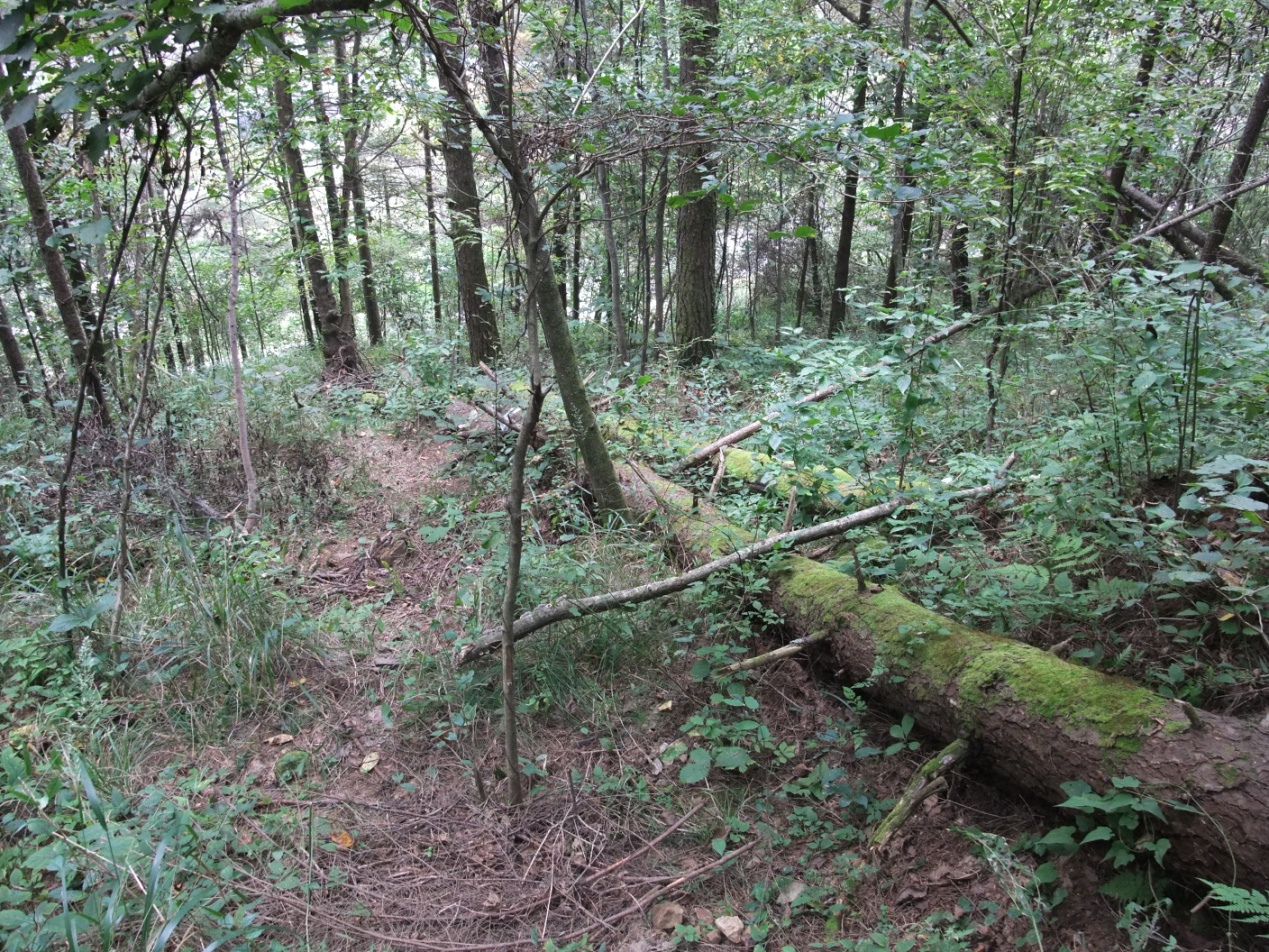

Supplement: S2 Table — (DOCX) [file pone.0175203.s002.docx]
